# Rare Somatic MEN1 Gene Pathogenic Variant in a Patient Affected by Atypical Parathyroid Adenoma

**DOI:** 10.1155/2020/2080797

**Published:** 2020-04-27

**Authors:** Luigia Cinque, Flavia Pugliese, Celeste Clemente, Stefano Castellana, Maria Pia Leone, Danilo de Martino, Teresa Balsamo, Claudia Battista, Tommaso Biagini, Paolo Graziano, Marco Castori, Alfredo Scillitani, Vito Guarnieri

**Affiliations:** ^1^Division of Medical Genetics, Fondazione IRCCS Casa Sollievo Della Sofferenza, San Giovanni Rotondo (FG), Italy; ^2^Unit of Endocrinology, Fondazione IRCCS Casa Sollievo Della Sofferenza, San Giovanni Rotondo (FG), Italy; ^3^Unit of Pathology, Fondazione IRCCS Casa Sollievo Della Sofferenza, San Giovanni Rotondo (FG), Italy; ^4^Bioinformatic Unit, Fondazione IRCCS Casa Sollievo Della Sofferenza, San Giovanni Rotondo (FG), Italy; ^5^Unit of General Surgery 2nd and Thoracic Surgery, Fondazione IRCCS Casa Sollievo Della Sofferenza, San Giovanni Rotondo (FG), Italy; ^6^Laboratory of Oncology, Fondazione IRCCS Casa Sollievo Della Sofferenza, San Giovanni Rotondo (FG), Italy; ^7^Bioinformatic Unit, Istituto Mendel-CSS, Rome, Italy

## Abstract

**Objective:**

Atypical parathyroid adenoma is a rare neoplasm, showing atypical histological features intermediate between classic benign adenoma and the rarest parathyroid carcinoma, whose the clinical behaviour and outcome is not yet understood or predictable. Up to date only two cases of atypical adenoma were found associated to a MEN1 syndrome, and only one was proved to carry a pathogenic variant of the MEN1 gene.

**Design:**

We report the clinical, histologic, and molecular findings of a 44-year-old woman, presenting with a histologically proved atypical parathyroid adenoma with an apparent aggressive behaviour.

**Methods and Results:**

CDC73 gene was screened at germline and somatic levels with no results. Whole exome sequencing performed on DNA extracted from blood leukocytes and tumour tissue revealed a somatic MEN1 gene heterozygous variant, c.912+1G > A, of the splicing donor site of exon 6. On immunohistochemistry, downregulation of the menin protein expression in the neoplastic cells was also observed.

**Conclusions:**

We report the second case of a rare association of a somatic MEN1 gene mutation in a patient with atypical parathyroid adenoma. We suggest that MEN1 gene could be an underestimate genetic determinant of these rare histological entities, and we highlight the utility of a complete genetic screening protocol, by the use of next-generation sequencing technology in such undetermined clinical cases with no frank clinical presentation.

## 1. Introduction

Primary hyperparathyroidism (pHPT) is the third most common endocrine disease after diabetes and thyroid disorders [1]. It is characterized by hypercalcemia sustained by inappropriate high parathyroid hormone (PTH) levels, the latter due to a parathyroid neoplasm. Benign parathyroid adenoma (PA) is the main common cause of pHPT in up to 85% of cases, and the remaining 15% by hyperplasia [1]. Less than 1% of cases includes the rare parathyroid carcinoma (PC), while an intermediate histology entity, between the PA and the PC, defined as atypical adenoma (AA) has been reported with a frequency ranging from 0.5% to 4.4% with highest frequency for the selected ethnic group [1, 2]. Recognizing both PC, especially in absence of metastases, and AA is not clear-cut in all cases. The PC presents with severe hypercalcemia (>14 mmg/dL), with a parathyroid lesion of 3 cm in average (from 2 to 10 g) [3] with a firm adherence and invasion of surrounding structures [4]. In 70% of sporadic PC cases, as well as in 15–20%, associated to hyperparathyroidism with jaw tumour syndrome (HPT-JT), pathogenic variants of the CDC73 gene were found, with the corresponding loss of expression of the encoded parafibromin protein, on the tumour tissues [5–7].

In fact, in absence of a well-defined vascular invasion and/or metastasis, differential diagnosis between AA and PC might be challenging. Histological criteria observed in AA are represented by cellular atypia together with solid growth pattern, fibrous septa, and adherence to surrounding thyroid tissue, with or without the so called capsule pseudoinvasion [8]. Moreover, no specific molecular markers are available, taking into account that, despite many efforts spent in this specific field, a handful of pathogenic CDC73 variants have been reported in AA [9–11].

Thus, the AA can be considered a challenge not only for the expert pathologist but also for the endocrinologist. Because of the rarity, its natural history is unknown, postsurgical management of these patients is not predictable, and few anecdotal information are available about the outcome [12, 13]. Whether, in terms of origin and prognosis, AA is more similar to PA remains likewise to be evaluated: in this case, a possible molecular marker could be the MEN1 gene, that results mutated in the namesake syndrome, characterized by familial HPT, benign parathyroid adenoma or hyperplasia, gastro-entero-pancreatic and pituitary tumors (the latter 40% and 30% of cases, respectively) and less frequently by adrenocortical and thyroid carcinoids and lipomas [14, 15]. Interestingly, although in MEN1 pHPT is almost always associated with benign parathyroid lesions, adenoma, or hyperplasia [1, 2], mutations of the gene have been found in 35% of PA and 6% of PC [16–18], but also in a small series of AA, in 1 out of 10 cases (10%) [2]. This could suggest a wider presentation of parathyroid disease in this condition.

We report a 44-year-old woman with pHPT and severe hypercalcemia due to AA. Whole exome sequencing (WES) revealed a previously known MEN1 variant that occurred somatically in the parathyroid lesion.

## 2. Material and Methods

### 2.1. Clinical History

An apparently healthy 44-year-old woman was admitted for recurrent bilateral renal colic, with an ultrasound picture of bilateral renal stones with hydronephrosis. The blood chemistry performed for diagnostic analysis showed a picture of primary hyperparathyroidism with severe hypercalcemia (14 mg/dL, n.r. = 8.4–10.2 mg/dL) and very high parathormone values (1347 pg/mL, n.r. = 10–65 pg/mL). On neck ultrasound, a richly vascularized hypoechoic parathyroid lesion of 1.5 cm in diameter was detected inferiorly to the lower left thyroid pole. Due to high levels of serum calcium, she was hydrated with 4 L of intravenous physiological solution and 3 L of water “per os.” However, normocalcaemia was not reached, and 4 mg of intravenous zoledronic acid was administered. She underwent surgery with removal of the lower left parathyroid and left thyroid lobe, with macroscopic histological finding of parathyroid carcinoma. After parathyroidectomy, serum calcium levels returned to normal and no biochemical or instrumental signs of recurrence have been observed to date (10 years of follow-up).

### 2.2. Molecular Study

After informed consent, DNA was extracted from peripheral blood and the protocol was approved by our local ethic committee (*Prot-Familia-*16/CE/2016). The work was done according to the 1984 Helsinki declaration and its subsequent versions.

Due to the severity of the biochemical profile and the original histological suspect, Sanger sequencing for both the CDC73 and MEN1 genes and the search for large deletions at the CDC73 locus were attempted [19]. Then, in search of a possible alternative genetic explanation, WES was performed on DNA extracted from blood and paraffin-embedded tumoral lesions. Briefly, gDNA concentration was determined with the Qubit dsDNA BR Assay (Invitrogen, Carlsbad, CA, USA), and the fluorescence was measured using the Qubit Fluorometer (Invitrogen, Carlsbad, CA, USA). A total of 50 ng of gDNA was used for library preparation. The library was prepared using Agilent SureSelect QXT Clinical Research Exome kit (Agilent Technologies, Santa Clara, CA), according to SureSelect QXT Target Enrichment for Illumina Multiplexed Sequencing protocol. gDNA was enzimatically fragmented, and adaptors were ligated to the DNA fragments. The fragments were purified with AMPure beads and PCR amplified. The library was used in the hybridization and captured with the biotinylated SureSelect bait. The SureSelect-enriched DNA libraries were purified according to the manufacturer's recommendations and PCR amplified using an appropriate pair of dual indexing primers. The amplified indexed DNA products were checked for quality (2200 Tape Station and High Sensitivity D1000 ScreenTape, Agilent Technologies, Santa Clara, CA), normalized, pooled, and sequenced using the NextSeq500 platform (Illumina, San Diego, CA) at an average coverage depth of 70X. *In silico* analysis was carried out comparing variants found on constitutional and somatic DNAs (i.e., occurring in the paraffin-embedded lesion, but absent in the peripheral blood). In particular, genes affecting the parathyroid function and neoplasms, CDC73, MEN1, CDKN1B, and GCM2, were screened.

The presence of the variant was confirmed by Sanger sequencing on somatic DNA: the PCR was carried out to amplify both exons 5 and 6 (which located close to each other) of the MEN1 gene, using the following forward 5′-tgcccgataggctaaggac-3′ and reverse 5′-actgttagggtctcccttct-3′ primers. The PCR product was purified (ExoSAP-IT, Thermo Fisher Scientific, Waltham, MA, USA) and sequenced (ABI Prism 3100 Genetic Analyzer, Thermo Fisher Scientific Waltham, Massachusetts, USA) using the BigDye Terminator v1.1 sequencing kit (Applied Biosystems, Foster City, CA, USA).

### 2.3. Immunohistochemistry

3-*μ*m thick representative parathyroid tumor tissue sections were deparaffinized in xylene, rehydrated in graded alcohols, washed in double-distilled water, and treated with DAKO solution (EnVision FLEX Target Retrieval Solution) for antigen retrieval. The slides were treated with primary anti-menin monoclonal antibody (clone B-9, diluted 1 : 200, Santa Cruz Biotechnology) for 30 min. Antigen-antibody reaction was visualized using the EnVision FLEX kit (Dako Agilent) with diaminobenzidine as chromogen. After counterstaining with hematoxylin, the slides were covered.

## 3. Results

### 3.1. Molecular Findings

**Figure 1:**
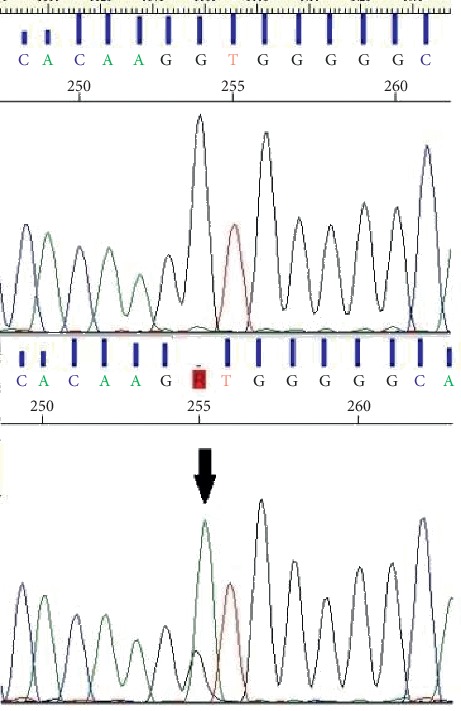
Electropherograms showing the splicing pathogenic variant G > A (arrow). On the top: germline DNA; on the bottom: somatic DNA.

We did not find any deleterious germline variants in both the CDC73 and MEN1 genes. Conversely, at the somatic level, the WES revealed a previously reported MEN1 gene splicing nucleotidic change, namely, c.912+1G > A, in heterozygosity, affecting the donor site of exon 6 and predicted to cause the skipping of exon 6 with the insertion of a premature stop codon, p.(Tyr276Arg ∗ 62) (see [14] and reference therein). As stated above, the variant was not present on germline DNA (Figure 1), thus confirming the sporadic nature of the clinical case.

### 3.2. Pathologic Findings and IHC for MEN1 Protein

Grossly, surgical specimen consisted of left thyroid lobe strictly adhering to a solid, capsulated parathyroid neoplasm (1.5 cm in diameter).

**Figure 2:**
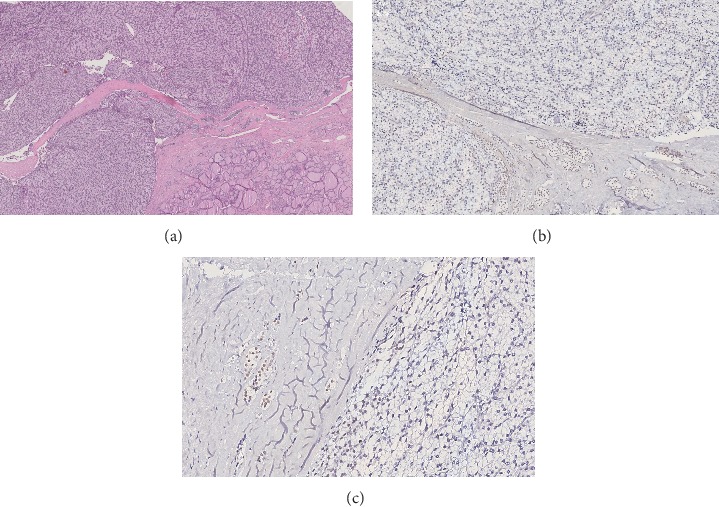
(a) Parathyroid adenoma: on hematoxylin-eosin tissue sections, the pushing growth pattern of the tumor and the strict adherence to thyroid tissue is observed. (b). Reduction of nuclear immunoreactivity of menin protein was observed in the neoplastic cell. (c). High-power view (X30) highlights decreased menin expression in neoplastic cells, whereas entrapped parathyroid normal cells maintain strong nuclear menin immunoreactivity.

Microscopically, parathyroid lesions showed a trabecular growth pattern and thick fibrous septa and separated solid nests of clear tumor cells. Moreover, some aggregates of neoplastic cells entrapped in the context of the tumor capsule were observed, and focal infiltration of thyroid tissue was also identified (Figure 2(a)). Neither obvious mushroom-like infiltration of the capsule nor vascular invasion was demonstrated. Mitoses were present in a number less than 1 per 10 high-power field.

While positivity for parafibromin antibody was observed (data not shown), menin immunoreactivity was reduced in neoplastic cells (Figures 2(b) and 2(c)).

## 4. Discussion

To our knowledge, the case here presented is the second reporting an AA due to a MEN1 gene mutation. AA is a histology entity thought to be located in an intermediate position between the more aggressive and rare PC and the common PA. Due to its rarity, the lack of information about the origin, the potential malignity and, mostly, of the possible outcome, make the AA a challenging tumor to deal with, that requires a strict follow-up. Several molecular attempts have been made in search of possible genetic or histological determinants for a better classification, recognition, and prognosis; however, these studies were not definitive, since they confirmed the partial overlapping with PC and PA. The CDC73 gene, the main gene of sporadic and familial PC, resulted mutated only in few cases of AA [9–11], and this initially suggested that the AA may be an intermediate progression step towards the full malignity. However, metanalyses on the use of the parafibromin protein expression and on the follow-up of patients with AA and CDC73 deleterious variants provided controversial results (see [2] and references therein). Conversely, involvement of MEN1 was not systematically investigated in AA. This because MEN1 is only rarely mutated in malignant parathyroid lesions (∼0.5%) [17, 20, 21], since, on the contrary, the CDC73 gene is considered the candidate gene for parathyroid lesions of uncertain malignity. Up to date, only two cases of AA were associated to MEN1 syndrome, and only one a germline mutation was also identified [2, 17]. However, at variance with our case who presented only nephrolythiasis and hypercalcemia associated to the parathyroid lesion, previously a clear landscape of clinical MEN1 syndrome was recognizable, being the patients affected by other related tumors, such as pancreas, adrenal, or thymic carcinoids (Table 1).

The case here showed is instructive for the following reasons: (i) the clinical presentation featured by severe hypercalcemia as well as the subsequent macroscopic observation at the surgery of the parathyroid lesion; both were evocative of a more aggressive disease (HPT-JT or sporadic PC); (ii) although the subsequent pathologic diagnosis lowered the malignant grade from PC to AA, it still prompted to search for a CDC73 gene involvement, resulted negative (along with the MEN1 gene), at germline level; and (iii) high-throughput technologies are now available at relatively low price and represent the good choice to gain the molecular answer for not clear-cut cases as the one here reported.

However, the limit of our work is related just to the rarity of AA: whether our finding would suggest that also the MEN1 gene can play a role in the onset of the AA, at the same time, it would will be hard to be confirmed due to the paucity of samples worldwide available, although the AA is more common that the rarest PC.

We confirmed that, in the absence of any reliable histological or molecular signature, the management of individuals with a previous AA is based on periodic clinical assessment. However, we believe that an extensive molecular testing including all known genes associated with parathyroid hyperplastic/neoplastic disorders [22–24] might support the clinicians in the management of these patients. This study, in particular, demonstrates that next generation sequencing technologies are helping in translating high-throughput molecular screening on both blood and paraffin-embedded lesions into clinical practice.

## Figures and Tables

**Table 1:** Clinical features of the three subjects reported in the literature with MEN1 syndrome and AA.

	Sex	Age	Ca^a^	PTH^b^	Parathyroid	Nephrolythiasis	MEN1 mutation	Other MEN1-related tumors	Others	Ref.
ID1	M	50	3.22 nmol/L	215 pmol/L	2 × 2.9 cm, 14 g	Bilateral	c.253A>T	Acromegalia, pituitary and adrenal, lipomas	CKD	Christakis et al. [18]

ID2	F	32	12 mg/dL	NA	0.7 cm	No	NA	Pancreas and thymus	No	Pal et al. [20]

ID3	F	44	14 mg/dL	1347 pg/mL	2 cm	Bilateral	c.912+1G>A	No	No	Present work

^a^Normal range: 1.12–1.32 mmol/L or 8.4–10.2 mg/dL; ^b^normal range 15–65 pg/mL; CKD = chronic kidney disease.

## Data Availability

This work does not include data that strictly require to be published in a public database.
